# The Importance of Biodiversity E-infrastructures for Megadiverse Countries

**DOI:** 10.1371/journal.pbio.1002204

**Published:** 2015-07-23

**Authors:** Dora A. L. Canhos, Mariane S. Sousa-Baena, Sidnei de Souza, Leonor C. Maia, João R. Stehmann, Vanderlei P. Canhos, Renato De Giovanni, Maria B. M. Bonacelli, Wouter Los, A. Townsend Peterson

**Affiliations:** 1 Centro de Referência em Informação Ambiental (CRIA), Campinas, São Paulo, Brazil; 2 Departamento de Micologia, Universidade Federal de Pernambuco (UFPE), Recife, Pernambuco, Brazil; 3 Departamento de Botânica, Universidade Federal de Minas Gerais (UFMG), Belo Horizonte, Minas Gerais, Brazil; 4 Departamento de Política Científica e Tecnológica (DPCT), Universidade Estadual de Campinas (UNICAMP), Campinas, São Paulo, Brazil; 5 Zoological Museum, University of Amsterdam, Amsterdam, The Netherlands; 6 Biodiversity Institute, University of Kansas, Lawrence, Kansas, United States of America

## Abstract

Addressing the challenges of biodiversity conservation and sustainable development requires global cooperation, support structures, and new governance models to integrate diverse initiatives and achieve massive, open exchange of data, tools, and technology. The traditional paradigm of sharing scientific knowledge through publications is not sufficient to meet contemporary demands that require not only the results but also data, knowledge, and skills to analyze the data. E-infrastructures are key in facilitating access to data and providing the framework for collaboration. Here we discuss the importance of e-infrastructures of public interest and the lack of long-term funding policies. We present the example of Brazil’s *species*Link network, an e-infrastructure that provides free and open access to biodiversity primary data and associated tools. *Species*Link currently integrates 382 datasets from 135 national institutions and 13 institutions from abroad, openly sharing ~7.4 million records, 94% of which are associated to voucher specimens. Just as important as the data is the network of data providers and users. In 2014, more than 95% of its users were from Brazil, demonstrating the importance of local e-infrastructures in enabling and promoting local use of biodiversity data and knowledge. From the outset, *species*Link has been sustained through project-based funding, normally public grants for 2–4-year periods. In between projects, there are short-term crises in trying to keep the system operational, a fact that has also been observed in global biodiversity portals, as well as in social and physical sciences platforms and even in computing services portals. In the last decade, the open access movement propelled the development of many web platforms for sharing data. Adequate policies unfortunately did not follow the same tempo, and now many initiatives may perish.

## Abstract 2

This Community Page calls for new governance and funding models to ensure the sustainability of national e-infrastructures—important for global systems and crucial for local education, research, and policy.

## The Importance of Biodiversity E-infrastructures for Informed Decision Making

Biodiversity e-infrastructures not only increase access to and usability of data for science, but also support education and effective public policies. This point is particularly true regarding sustainable development, in which a paucity of relevant data about species’ numbers, distributions, and status hinders and biases key biodiversity metrics [1]. For megadiverse countries like Brazil, free and open access to research-grade data in usable formats is crucial to addressing such challenges and making informed decisions and policies [2]. However, for users to be able to rely on information systems, it is crucial for them to operate with uninterrupted, long-term funding. The greater the size and complexity of a system, the higher the costs required to sustain it, but also the greater its potential value. This point holds for all infrastructures, be it a railway or an information system. E-infrastructures require long-term maintenance and constant development, continuous and dynamic evaluation and planning, and efficient governance models to assure continuity of the network and its services; otherwise, they perish just as would their physical counterparts [3].

In addressing conservation and sustainable development, policies and actions must be able to scale from local to global. Too many important decisions directly linked to biodiversity conservation, such as choice of sites for protected areas and assessment of species’ conservation status, or that affect conservation directly, such as the construction of roads, hydroelectric dams, and mining activities, are based on partial information or even on criteria unrelated to the objectives of the planned decision [2,4,5]. The massive global infrastructure expansion that has been occurring in the last decades and is expected to take place until 2050 can damage the environment beyond repair [5]. Many of these infrastructures will be built in developing countries that hold most of the planet’s biodiversity, emphasizing the need for relevant data and decision-making tools to be at hand [5]. It is estimated that 25 million kilometers of new roads will be built up until 2050, and the initial construction of roads in forested areas often sets off a chain of serious problems, including habitat fragmentation, illegal mining, and land speculation [5].

Every country should commit to increase its knowledge base to strengthen local, national, and global e-infrastructures by making data and information openly available (a commitment of the Biodiversity Convention, Article 17, and many other international agreements). Effective thematic and geographically delimited e-infrastructures provide a framework for networking and collaboration among data providers and users. Within this environment, shared data and knowledge are more likely to be used, not only for scientific development, but also in policies and decision support systems [6]. These e-infrastructures often reflect and respond directly to local challenges, besides feeding data and tools into global systems. The effective integration of science communities can be appreciated, for instance, in the formation of the VertNet initiative, which links North American institutions with vertebrate holdings [7]. Frequently, such communities collaborate in overcoming important challenges, such as georeferencing of large datasets. The outcome is a storehouse of primary, research-grade data, associated tools and knowledge that are useful from local to global scales, and a well-knit collaborative network of scientists.

## Brazil’s *species*Link Network

Such e-infrastructures are a reality in Latin America and include Comissión Nacional para el Conocimiento y uso de la Biodiversidad (CONABIO), a permanent interdepartmental commission, created in 1992 in Mexico; Instituto Nacional de Biodiversidad (INBio), a nongovernmental organization founded in 1989 in Costa Rica; and Humboldt Institute (Instituto de Investigación en Recursos Biológicos Alexandre Von Humboldt), a nonprofit civil organization of public nature, under the Ministry of Environment and Sustainable Development, created in 1993 in Colombia—each with different strategies and experiences but all openly sharing biodiversity data locally and with Global Biodiversity Information Facility (GBIF). All three institutions have more than 20 years of history and services, but whereas CONABIO as a governmental institute and Humboldt, linked to the Ministry of Environment, have full support, INBio is struggling to survive [8].

**Fig 1:**
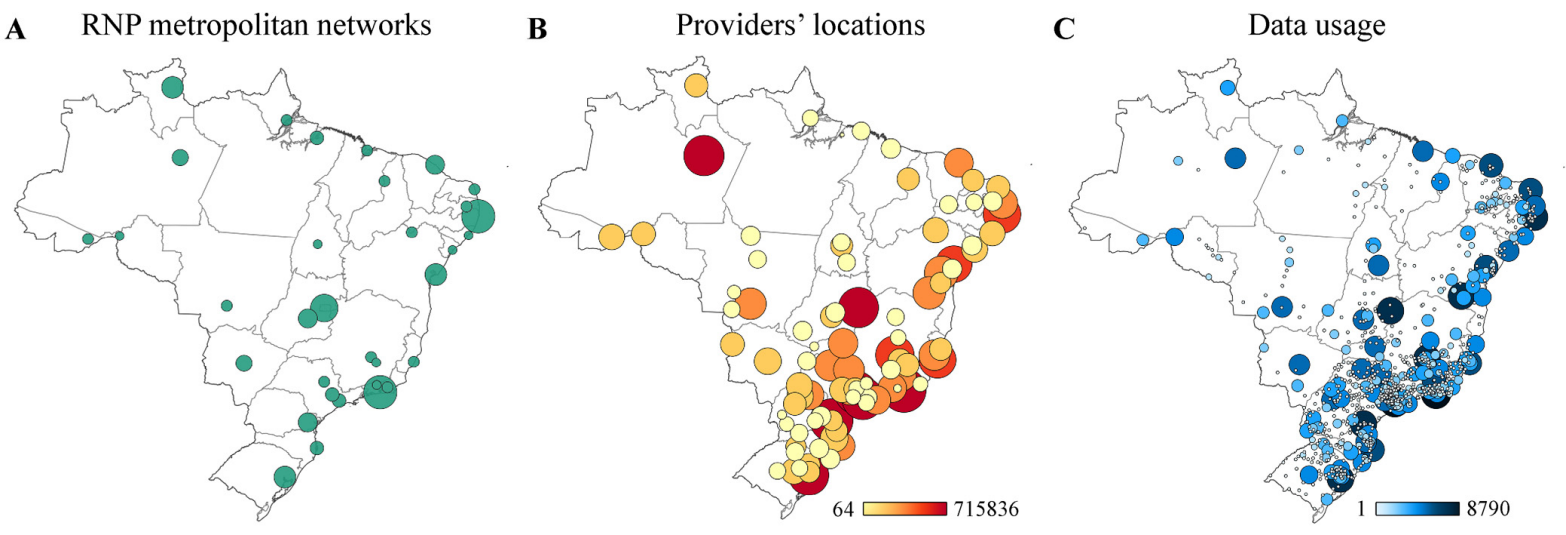
Research infrastructure and biodiversity data usage in Brazil. (A) Distribution of Rede Nacional de Ensino e Pesquisa (RNP) metropolitan networks (December 2014). (B) Distribution of *species*Link’s data providers (per institution) and amount of records shared (December 2014). (C) *species*Link data usage (sessions) across Brazil (2014). *Image credit*: *Eduardo G*. *Baena*.

Centro de Referência em Informação Ambiental (CRIA) was established in 2000 as a nonprofit association with the aim of disseminating scientific information and in this way contributing to the conservation and sustainable use of Brazil's biological resources. To fulfill its mission, CRIA is responsible for the development of *species*Link, a major biodiversity e-infrastructure conceived in 2001 within the Biota-FAPESP Program-The Virtual Institute of Biodiversity, focused on the biodiversity of São Paulo state, and later expanded to the entire country thanks to funding from Brazil’s Ministério da Ciência, Tecnologia e Inovação and to the involvement of scientific societies and biological collections. Currently, *species*Link provides free and open access to ~7.4 million primary, research-grade, biodiversity data records from 382 datasets from 135 national institutions, covering all Brazilian states (Fig 1B), and from 13 institutions from abroad.

Besides the open dissemination of data and tools, as most biodiversity e-infrastructures rely on a great number of data providers, an important output of these systems is the establishment of social networks, where each node has an important role to play. In the case of *species*Link, each data provider is responsible for digitizing, updating, and improving its data quality and has full control over its data. RNP is responsible for Brazil’s National Research and Education Network, which links more than 400 institutions through 41 metropolitan networks and hosts all *species*Link’s equipment and servers, guaranteeing data security, access, and uninterrupted operation. CRIA is responsible for maintaining and continuously developing the information system, attending the demands of both data providers and users.

All components of the network—data providers, users, RNP, and CRIA—are equally important. The strength of this collaborative network lies in integrating biological collections associated with research institutions and graduate programs, researchers, decision makers, software developers, and many other actors, all responsible for the intensive development, sharing, and usage of data and tools (S1 Text). In 2014, *species*Link had an average of 1.4 million records retrieved per day (S1 Table). About 95% of the access to the network is from Brazil, and its distribution is strongly correlated with the metropolitan networks and location of data providers (Fig 1A and 1C).

## The Brazilian Virtual Herbarium of Flora and Fungi

**Fig 2:**
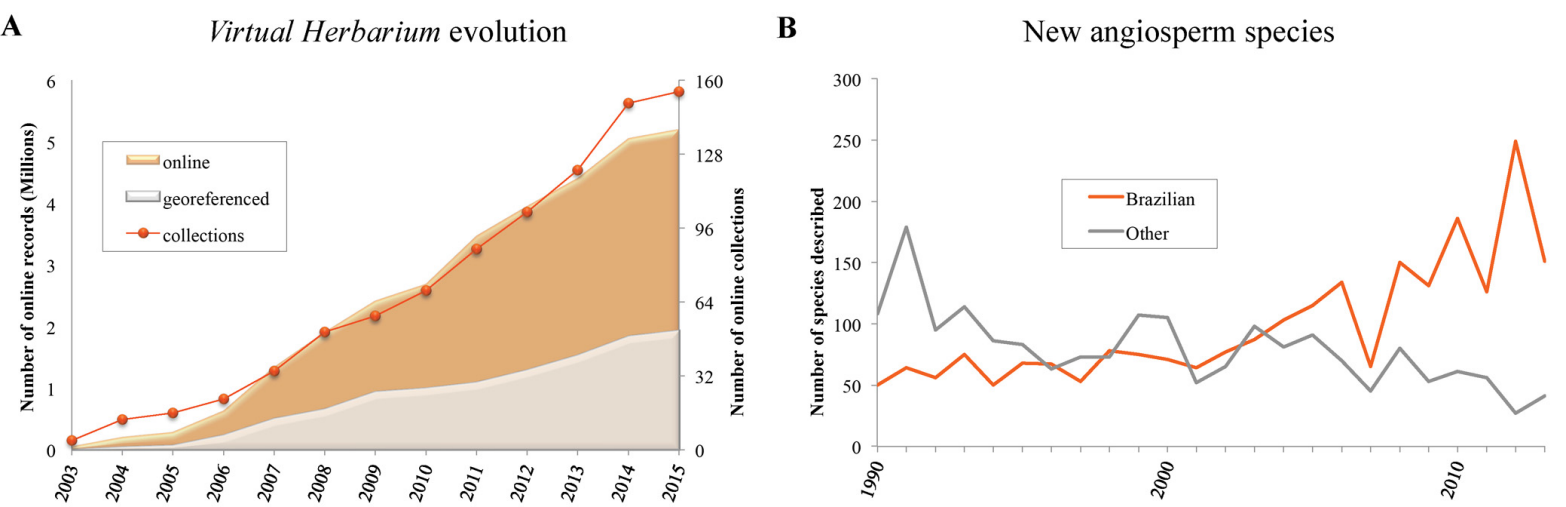
The evolution of Brazilian flora primary data available online and species described by Brazilian specialists. (A) Growth of the Virtual Herbarium from May 2003 to Feb 2015, showing the monthly average of online and georeferenced records; the orange line shows the evolution of the number of datasets. (B) Number of angiosperms species described by Brazilian (orange line) and foreign (grey line) scientists from 1990 to 2013.

One of Brazil’s Institutos Nacionais de Ciência e Tecnologia, the Brazilian Virtual Herbarium of Flora and Fungi, is one of *species*Link’s thematic nodes. Currently 54% of Brazil’s active herbaria participate in the network, thanks to the coordinated effort of the Virtual Herbarium and support from the Conselho Nacional de Desenvolvimento Científico e Tecnológico (CNPq). The marked increase of online data in Brazil’s Virtual Herbarium results from the Brazilian government’s investment in training human resources in taxonomy and curation since 2005 (Protax–CNPq) and in its support to the Virtual Herbarium between 2009 and 2014 (Fig 2A). This investment also accelerated the description of new species of plants and fungi between 2002 and 2013, when 2,372 new species of flowering plants were described [9] (updated to 2013 using The International Plant Names Index [IPNI] as the data source). More important is the apparent correlation between the increased rate of species description with the availability of online data and the fact that in the last decade 74% of new species were described by taxonomists based in Brazil (Fig 2B). These numbers are strong indications that Brazil is beginning to reap the benefits of investments in taxonomy and that access to information, tools, and knowledge and the networking environment translates into academic power, which in turn enables the use of data for informed decision making. For example, in 2013, *species*Link data was used to produce a new version of Brazil’s Red List of Plants by the Centro Nacional de Conservação da Flora under the coordination of the Ministério do Meio Ambiente [10].

The large number of online records associated with the continuous update of the List of Species of the Brazilian Flora (http://floradobrasil.jbrj.gov.br) has also enabled the development of new analytical tools such as *Lacunas* and BioGeo. *Lacunas* helps identify gaps of data and knowledge in the Virtual Herbarium for different taxonomic groups (S1 Fig, S2 Table, and S3 Table) [11], while BioGeo facilitates the generation of distribution models of Brazilian plant and fungus species through a predefined workflow (S2 Fig) [12]. Both systems are available through public interfaces and were specifically developed to facilitate the design of research and collection strategies and to support planning and monitoring of conservation policies.

## The Need for Long-Term Funding for Biodiversity E-infrastructures

Part of the problem that e-infrastructures have been facing is that agencies prioritize short-term innovative projects and rarely fund ongoing and long-term initiatives [13]. Options envisioned to ensure stable funding include creation of dedicated grants by funding agencies [13], the integration of e-infrastructures into larger collaborative information systems [3], and the creation of new independent public agencies to guide the work of producing, evaluating, and updating a formal legal framework for e-infrastructures’ governance [14]. In 2009, the European Research Infrastructure Consortium was created to provide a new legal support system for European research infrastructures, highlighting the importance of new governance frameworks that provide lasting arrangements for secured funding [15]. A recent study analyzing 16 e-infrastructures across different disciplines such as physics, biology, and computing sciences showed that only six had reached sustainable funding, while nearly half lacked long-term funding [15]. Even global projects such as Encyclopedia of Life and Barcode of Life [16] were imperiled in the past, because they simply ran short of funding or the time to meet their aims was miscalculated and the money was not enough to accomplish all goals.

From the outset, *species*Link has been sustained through project-based funding, normally public grants for 2–4-year periods. Each new project promotes new developments and attracts new data providers and users but also increases the demand for support services in the long-term. In between projects, there are short-term crises in trying to keep the system operational. A strategy to consolidate a biodiversity e-infrastructure in Brazil was developed by three scientific societies in 2005 under the coordination of the Ministério da Ciência, Tecnologia e Inovação [17]. An essential element of the plan was the expansion and further development of *species*Link to include new partners, more collections, and developers, establishing a truly comprehensive national network. This plan has guided most developments of *species*Link and was the basis for a component of the Global Environment Facility–funded project to establish the Sistema de Informação sobre a Biodiversidade Brasileira, launched in November 2014. Yet, a strategy for long-term funding has not materialized. Quite simply, no public mechanisms exist to fund the maintenance and continuous development of such systems of public interest so, in spite of *species*Link’s success, scientists and developers are struggling to keep the initiative alive.

The time has come for countries to seriously support biodiversity information e-infrastructures: some must support initial steps in implementation, whereas other countries (e.g., Brazil, Mexico, Colombia, Costa Rica, and many others) have the luxury of having facilities already in place. If effective steps to secure the permanence of e-infrastructures are not taken soon, we will risk having biological data, which are currently organized and made available globally, once again inaccessible. In the case of Brazil, *species*Link is in immediate peril of disappearing. Brazil is one of the most diverse countries in the planet [18], holding ~19% of all existing plant species [19]; thus, *species*Link is not only of interest to Brazilian people and government anymore but has acquired importance in the global scenario as well. Not only will the hundreds of thousands of users of this system miss this crucial research and policy infrastructure, but the social scientific network linked to the e-infrastructure may lose strength.

## Supporting Information

S1 Fig *Lacunas* workflow. *Lacunas* is a system designed to help identify knowledge and information gaps, based on online data available in Brazil’s Virtual Herbarium of Plants and Fungi (http://lacunas.inct.florabrasil.net). Besides data from Brazil’s Virtual Herbarium, other data sources include the List of Species of the Brazilian Flora and the country’s official Red List of Threatened Plants (Ministério do Meio Ambiente, 2014). Besides assessing each species’ data status, *Lacunas* enables an evaluation of data and information gaps per taxonomic group over time. This is an important indicator for data content of the Virtual Herbarium.
(TIF)

S2 Fig BioGeo workflow. BioGeo is a platform for e-science that uses ecological niche modelling techniques to improve the understanding of plant biogeography in Brazil (http://biogeo.inct.florabrasil.net). Potential distribution maps can be generated, searched, visualized, and downloaded for each species. The system is based on a workflow that uses a number of services and has instances that depend on expert opinion. As a result, through voluntary collaboration, >3,000 species now present geographic distribution models, and these models are available in the *Lacunas* report.
(TIF)

S1 Table Content and usage of *species*Link in 2013 and 2014. Table shows the total content and usage of *species*Link in years 2013 and 2014. Content is expressed as online records and images. Besides searching the database, the interface offers a number of tools or commands that can be triggered, such as listing the records, producing graphs and maps with the data, visualizing images, and downloading data. These numbers only refer to the user interface and do not include data provided through web services.
(DOCX)

S2 Table Percentage of species without records in the Brazil’s Virtual Herbarium (http://inct.splink.org.br). Comparing the total number of species without any online records in January 2013 (8,176 species, representing about 19% of all known species) with January 2015 (6,770, about 15% of all known species), one sees a clear qualitative evolution of the Virtual Herbarium. The increase of the number of species that have >20 occurrence points is another parameter that *Lacunas* presents, indicating the percentage of species that potentially can produce good ecological niche models. Search parameters: phonetic search by the accepted name of the List of Species of the Brazilian Flora, plus synonyms, including records with or without geographic coordinates.
(DOCX)

S3 Table Percentage of species with more than 20 distinct occurrences in the Brazil’s Virtual Herbarium (http://inct.splink.org.br). In January 2013, 6,712 species (15.6%) had distinct and consistent geographic coordinates; by 2015, this number went up to 8,473 species, or 18.5% of the total. This trend represents an increase of >26% of species that potentially can be used to develop ecological niche models, another important indicator of increase of digital knowledge. Search parameters: phonetic search by the accepted name of the List of Species of the Brazilian Flora, plus synonyms, including records with consistent and distinct geographic coordinates.
(DOCX)

S1 Text Details about *species*Link tools. (DOCX)

## Abbreviations

Biota-FAPESP Program: FAPESP Research Program on Biodiversity Characterization, Conservation, Restoration and Sustainable Use
CNPq: Conselho Nacional de Desenvolvimento Científico e Tecnológico
CONABIO: Comissión Nacional para el Conocimiento y uso de la Biodiversidad
CRIA: Centro de Referência em Informação Ambiental
GBIF: Global Biodiversity Information Facility
INBio: Instituto Nacional de Biodiversidad
IPNI: The International Plant Names Index
RNP: Rede Nacional de Ensino e Pesquisa
