# Supplementary figures and images for: The Importance of Biodiversity E-infrastructures for Megadiverse Countries
Source: PLoS Biol. 2015 Jul 23;13(7):e1002204. doi: 10.1371/journal.pbio.1002204 (PMC4512726; doi:10.1371/journal.pbio.1002204)

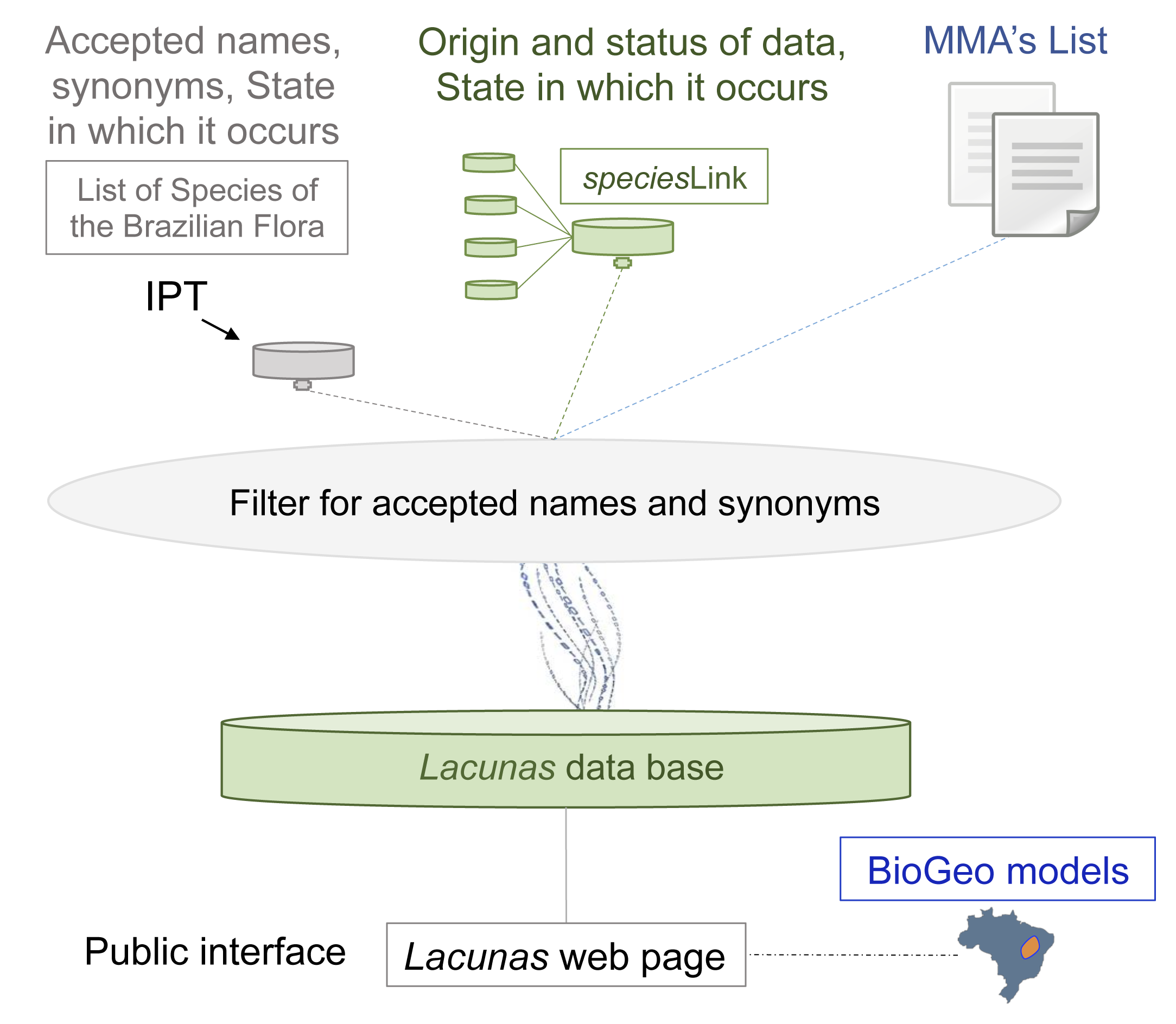

Supplement: S1 Fig — Lacunas is a system designed to help identify knowledge and information gaps, based on online data available in Brazil’s Virtual Herbarium of Plants and Fungi (http://lacunas.inct.florabrasil.net). Besides data from Brazil’s Virtual Herbarium, other data sources include the List of Species of the Brazilian Flora and the country’s official Red List of Threatened Plants (Ministério do Meio Ambiente, 2014). Besides assessing each species’ data status, Lacunas enables an evaluation of data and information gaps per taxonomic group over time. This is an important indicator for data content of the Virtual Herbarium. (TIF) [file pbio.1002204.s001.tif]

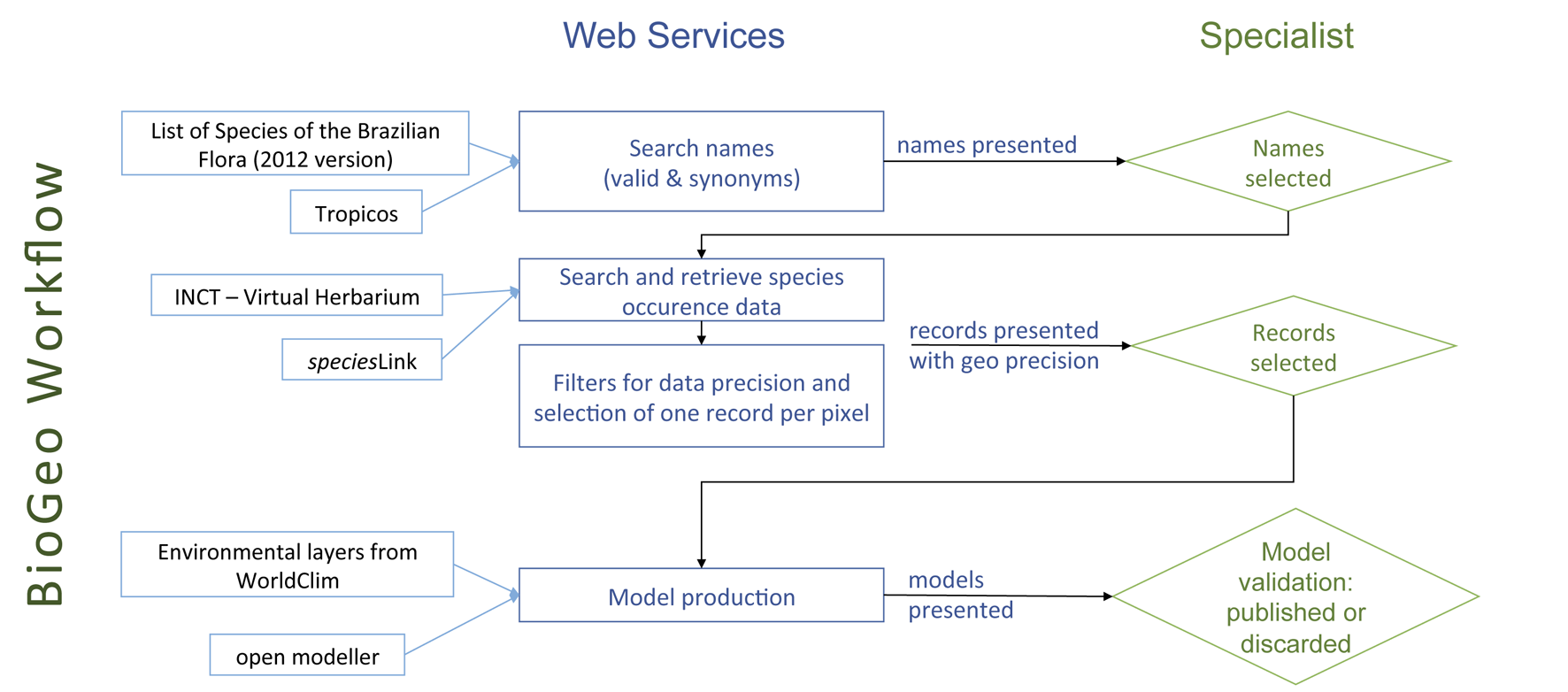

Supplement: S2 Fig — BioGeo is a platform for e-science that uses ecological niche modelling techniques to improve the understanding of plant biogeography in Brazil (http://biogeo.inct.florabrasil.net). Potential distribution maps can be generated, searched, visualized, and downloaded for each species. The system is based on a workflow that uses a number of services and has instances that depend on expert opinion. As a result, through voluntary collaboration, >3,000 species now present geographic distribution models, and these models are available in the Lacunas report. (TIF) [file pbio.1002204.s002.tif]
