# Supplementary material for: The Importance of Biodiversity E-infrastructures for Megadiverse Countries
Source: PLoS Biol. 2015 Jul 23;13(7):e1002204. doi: 10.1371/journal.pbio.1002204 (PMC4512726; doi:10.1371/journal.pbio.1002204)
Supplement: S1 Table — Table shows the total content and usage of speciesLink in years 2013 and 2014. Content is expressed as online records and images. Besides searching the database, the interface offers a number of tools or commands that can be triggered, such as listing the records, producing graphs and maps with the data, visualizing images, and downloading data. These numbers only refer to the user interface and do not include data provided through web services. (DOCX) [file pbio.1002204.s003.docx]

| **Item** | **2013** | **2014** | **Growth** |
| --- | --- | --- | --- |
| **Content** | | | |
| Total online records (December, Year) | 6,270,837 | 7,144,483 | 14% |
| Total number of images (December, Year) | 274,581 | 922,772 | 236% |
| **Usage** | | | |
| Number of searches | 232,390 | 460,066 | 98% |
| Total commands (search, list, graph, map, images, download) | 827,730 | 1,368,930 | 65% |
| Total records matching search criteria | 1,734,027,522 | 2,593,178,119 | 50% |
| Total records retrieved (visualized, downloaded) | 387,769,370 | 512,635,142 | 32% |
| Records retrieved per day | 1,089,240 | 1,439,986 | 32% |
| Records retrieved/total online records/year | 62 | 72 | 16% |
| Records retrieved/total online records/month | 5 | 6 | 16% |
