# Supplementary material for: The Importance of Biodiversity E-infrastructures for Megadiverse Countries
Source: PLoS Biol. 2015 Jul 23;13(7):e1002204. doi: 10.1371/journal.pbio.1002204 (PMC4512726; doi:10.1371/journal.pbio.1002204)
Supplement: S2 Table — Comparing the total number of species without any online records in January 2013 (8,176 species, representing about 19% of all known species) with January 2015 (6,770, about 15% of all known species), one sees a clear qualitative evolution of the Virtual Herbarium. The increase of the number of species that have >20 occurrence points is another parameter that Lacunas presents, indicating the percentage of species that potentially can produce good ecological niche models. Search parameters: phonetic search by the accepted name of the List of Species of the Brazilian Flora, plus synonyms, including records with or without geographic coordinates. (DOCX) [file pbio.1002204.s004.docx]

|  | **Jan/13** | | **Jan/14** | | **Jan/15** | | **2013-2015** |
| --- | --- | --- | --- | --- | --- | --- | --- |
| **Taxonomic groups** | **spp.** | **%** | **spp.** | **%** | **spp.** | **%** | **Comparison (%)** |
| **Algae** | 2517 | 59.7 | 2209 | 52.4 | 2418 | 51.3 | -3.9 |
| **Angiosperms** | 2371 | 7.5 | 1881 | 5.9 | 1715 | 5.2 | -27.7 |
| **Bryophytes** | 323 | 21.2 | 280 | 18.3 | 202 | 13.3 | -37.5 |
| **Fungi** | 2850 | 64.0 | 2689 | 60.4 | 2364 | 41.9 | -17.1 |
| **Gymnosperms** | 0 | 0.0 | 0 | 0.0 | 1 | 3.3 | - |
| **Pteridophytes** | 115 | 9.5 | 94 | 7.8 | 70 | 5.6 | -39.1 |
| **TOTAL** | 8176 | 18.9 | 7153 | 16.6 | 6770 | 14.8 | -17.2 |

.
