# Supplementary material for: The Importance of Biodiversity E-infrastructures for Megadiverse Countries
Source: PLoS Biol. 2015 Jul 23;13(7):e1002204. doi: 10.1371/journal.pbio.1002204 (PMC4512726; doi:10.1371/journal.pbio.1002204)
Supplement: S3 Table — In January 2013, 6,712 species (15.6%) had distinct and consistent geographic coordinates; by 2015, this number went up to 8,473 species, or 18.5% of the total. This trend represents an increase of >26% of species that potentially can be used to develop ecological niche models, another important indicator of increase of digital knowledge. Search parameters: phonetic search by the accepted name of the List of Species of the Brazilian Flora, plus synonyms, including records with consistent and distinct geographic coordinates. (DOCX) [file pbio.1002204.s005.docx]

|  | **Jan/13** | | **Jan/14** | | **Jan/15** | | **2013-2015** |
| --- | --- | --- | --- | --- | --- | --- | --- |
| **Taxonomic groups** | **spp,** | **%** | **spp,** | **%** | **spp,** | **%** | **Comparison (%)** |
| **Algae** | 60 | 1.4 | 47 | 1.1 | 100 | 2.1 | 66.7 |
| **Angiosperms** | 6147 | 19.4 | 6835 | 21.5 | 7675 | 23.4 | 24.9 |
| **Bryophytes** | 181 | 11.9 | 193 | 12.6 | 228 | 15.0 | 26.0 |
| **Fungi** | 14 | 0.3 | 13 | 0.3 | 20 | 0.4 | 42.9 |
| **Gymnosperms** | 5 | 20.0 | 5 | 20.0 | 5 | 16.7 | 0.0 |
| **Pteridophytes** | 305 | 25.3 | 334 | 27.7 | 445 | 35.9 | 45.9 |
| **TOTAL** | 6712 | 15.6 | 7427 | 17.2 | 8473 | 18.5 | 26.2 |
