# Supplementary material for: The Importance of Biodiversity E-infrastructures for Megadiverse Countries
Source: PLoS Biol. 2015 Jul 23;13(7):e1002204. doi: 10.1371/journal.pbio.1002204 (PMC4512726; doi:10.1371/journal.pbio.1002204)
Supplement: S1 Text — (DOCX) [file pbio.1002204.s006.docx]

**S1 Text. Details about *species*Link tools.**

**Tools to enable data access and sharing**

*Species*Link’s tools and architecture are designed to enable full participation of all data providers, regardless of hardware, software, communication, or expertise limitations. An early challenge was to set up a network where data providers with minimal IT support and poor Internet connectivity could have full control over the data, determining what is made openly and freely available, which software to use, and tools to update, add new data, or withdraw all data from the network. The list of data providers and their geographic distribution is available at <http://www.splink.org.br/showNetwork>.

*spLinker* (see more at <http://splink.cria.org.br/splinker>), is the software responsible for mapping data fields in accordance to DarwinCore data model and sending or deleting data from the cache nodes (see architecture at <http://splink.cria.org.br/architecture>).

**Data quality (**[**http://splink.cria.org.br/dc**](http://splink.cria.org.br/dc)**)**

Each dataset, when updated, runs through a set of applications that checks for errors and inconsistencies, producing a report with “suspect” records. All changes in the data must be made at its origin, by the providers, and resubmitted to the network.

**Indicators (**[**http://splink.cria.org.br/indicators**](http://splink.cria.org.br/indicators)**)**

Dynamic (or daily) reports are available online in the form of maps and based on online data only. This allows monitoring and evaluating each biological collection, taxonomic group, or the whole network.

**Search Interface (**[**http://www.splink.org.br**](http://www.splink.org.br)**)**

The search interface allows users to produce inventories, charts, maps, summaries, and to download the full dataset retrieved. Images can be compared, measured, and visualized in detail. Information from other sources, such as Encyclopedia of Life and Biodiversity Heritage Library, is integrated.

**Annotation System**

Users can send comments to the data provider and these comments remain publicly available. The annotation system launched in December 2012, has received 530 comments that are available online, associated to the respective record (January 22, 2015).
